# Incomplete obstructive diseases of the female genital tract: a classification analysis of 72 cases and a literature review

**DOI:** 10.3389/fmed.2026.1591734

**Published:** 2026-03-13

**Authors:** Hao Si, Changhong Zhang, Mei Yuan, Liu Dong, Huihui Jia, Zhihong Xie, Hui Wang

**Affiliations:** 1 Department of Obstetrics and Gynecology, The Affiliated Fuyang People’s Hospital of Anhui Medical University, Fuyang, Anhui, China; 2 Department of Obstetrics and Gynecology, The First Hospital of Xi'an, Xi'an, Shaanxi, China

**Keywords:** developmental abnormalities, disease classification, female genital tract, menstrual disorders, obstructive diseases, surgical treatment

## Abstract

**Objective:**

This study aimed to explore the diagnosis and treatment of patients who have reached menarche but present with clinical manifestations of genital tract obstruction.

**Methods:**

A retrospective analysis was conducted on 72 patients with abnormal menstrual blood discharge and genital tract developmental abnormalities treated at the Department of Obstetrics and Gynecology, the Affiliated Fuyang People’s Hospital of Anhui Medical University, from May 1994 to August 2024. The study evaluated the deformity features, clinical manifestations, treatment methods, and clinical outcomes, together with a literature review, and performed a classification analysis of female genital tract developmental abnormalities with incomplete obstructive clinical presentations.

**Results:**

(1) A total of 24 types of female genital tract developmental abnormalities were identified in patients who had attained menarche and presented with obstructive clinical presentations. (2) These diseases were collectively referred to as incomplete obstructive diseases of the female genital tract. (3) Incomplete obstructive diseases of the female genital tract were classified into four types based on the location of obstruction: incomplete vaginal orifice obstruction, incomplete vaginal obstruction, incomplete cervical obstruction, and incomplete uterine cavity obstruction.

**Conclusion:**

The classification and categorization of incomplete obstructive diseases of the female genital tract provide important clinical guidance for the diagnosis and treatment of patients who have attained menarche but have genital tract obstructive clinical manifestations.

## 1 Introduction

The incidence of congenital malformations of the female genital tract is approximately 4–7% (1), presenting with a variety of clinical manifestations due to differences in the location and extent of the abnormalities. A reliable and effective classification system is crucial for the clinical diagnosis and treatment of female genital tract developmental anomalies (2). Currently, the classification of congenital female genital tract developmental abnormalities (2, 3) remains controversial. Some scholars classify these abnormalities into obstructive and non-obstructive types based on whether menstrual blood flows freely (4–6). In clinical practice, some patients experience menarche but also present with dysmenorrhea or other obstructive clinical manifestations of the genital tract. These conditions are more frequently observed in adolescent female patients, and conventional treatment for dysmenorrhea or abnormal uterine bleeding is often ineffective. This study discusses the clinical diagnosis and treatment of such patients.

## 2 Materials and methods

### 2.1 Data source

Clinical data of 72 patients with genital tract developmental abnormalities and abnormal menstrual blood discharge who visited the Department of Obstetrics and Gynecology at The Affiliated Fuyang People’s Hospital of Anhui Medical University between May 1994 and August 2024 were included in this study (Table 1).

**Table 1:** List of cases of incomplete obstructive diseases of the female genital tract admitted to our hospital.

Disease	Number of cases (*n*)	Percentage (%)
Incomplete vaginal orifice obstruction:	45	62.50
Incomplete hymenal atresia	4	5.56
46,XX androgen excess type disorders of sex development	41	56.94
Incomplete vaginal obstruction:	24	33.33
Incomplete transverse vaginal septum	11	15.28
Oblique vaginal septum syndrome	13	18.05
Incomplete cervical obstruction:	2	2.78
Uterus didelphys with unilateral cervical atresia (type I) (13) (Oblique vaginal septum syndrome type IV) (10)	2	2.78
Incomplete uterine cavity obstruction:	1	1.39
Rudimentary horn of the uterus: type II	1	1.39
Sum up	72	100

This study was a retrospective analysis with a long data collection period (1994–2024). We acknowledge that, over such an extended time span, the level of detail and documentation format for physical examinations—particularly vulvar and vaginal examinations—varied across periods and physicians. To standardize as much as possible, all key physical examination findings reported in this article—e.g., hymenal morphology, the location of the vaginal septum, the status of the vaginal opening, and cervical visibility—were re-extracted from the original medical records and standardized or verified by senior gynecologists. Reporting in this study followed the CARE guidelines to ensure completeness of key information.

### 2.2 Diagnosis and treatment

**Figure 1:**
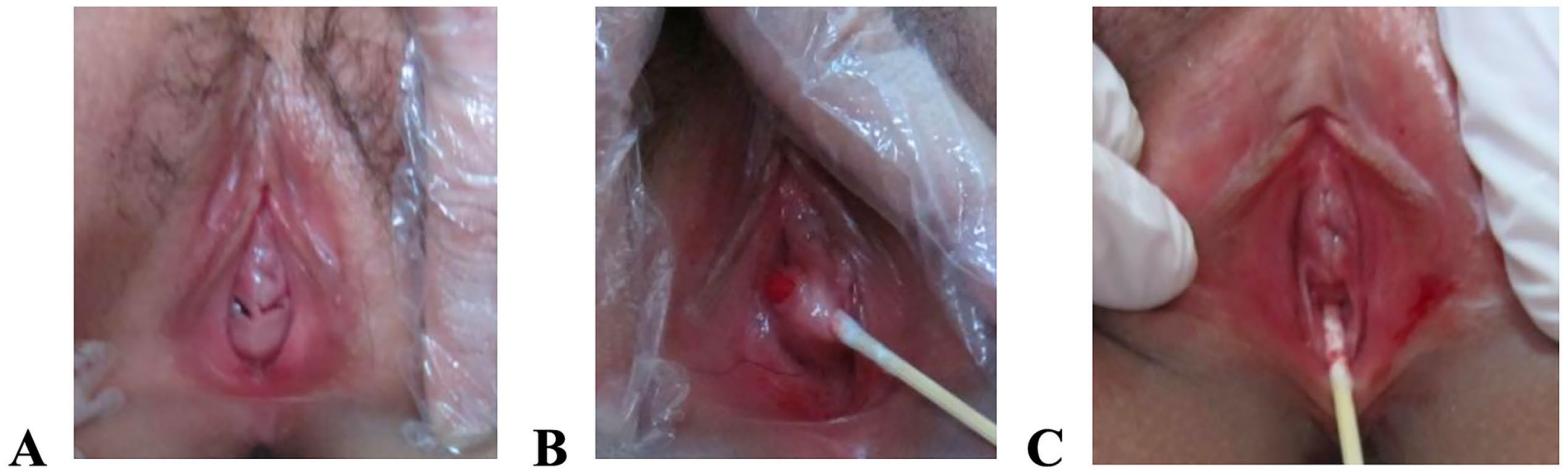
Schematic illustration of surgical correction for bridge-like hymenal atresia. **(A)** The labia majora and minora were normally developed; a bulge was present below the external urethral orifice, and two oblique fissures were located immediately adjacent to the lower margin of the external urethral orifice, from which dark-red blood flowed; **(B)** A throat swab probe revealed a membranous septum between the two fissures; **(C)** After incising the septum, the vaginal introitus appeared normal.

Four cases of incomplete hymenal atresia were identified, one of which had a bridge-like hymen. Due to the small size and concealed location of the two openings, the condition was initially misdiagnosed as the “absence of vagina” during early childhood. Following menarche in adolescence, a clear diagnosis was made in the outpatient department. Surgical incision of the hymen between the two small openings was performed, resulting in a successful outcome (7) (Figure 1).

In cases of disorders of sex development (DSD), 41 patients were diagnosed with female pseudohermaphroditism (FPH), also known as 46,XX androgen excess type DSD (8, 9). Family history and maternal medication history during pregnancy were carefully examined, and relevant laboratory and imaging tests were performed to establish the diagnosis. Based on the Prader classification and grading, surgical methods included vestibuloplasty, vaginoplasty, clitoral repositioning, labiaplasty, and urethroplasty with repositioning (7). One 23-year-old patient presented with a complex clinical condition: her mother had taken androgens during early pregnancy, resulting in external genital abnormalities at birth. At 14 years of age, she developed cyclical hematuria, and 5 years before her admission to the study hospital, she had undergone a genital correction surgery at another hospital. She was admitted for difficulty with intercourse after 2 months of marriage. Upon examination, the patient had fused labia that were partially open, with only a thin membranous septum separating the urethra and the vagina. The lower part of the urethra and its external opening were wide, suggesting that intercourse had occurred through the urethra. The patient underwent vaginoplasty, clitoral and urethral diameter reduction surgery, and urethral repositioning to prevent urethral syndrome, which involved mobilizing the external urethral opening and the lower urethra subcutaneously along the vestibular mucosa and fixing it upward.

A total of 11 cases of incomplete transverse vaginal septum were identified, including 8 cases of high-position incomplete transverse vaginal septum. Six of these cases were misdiagnosed: two were incorrectly diagnosed as cervical developmental anomalies at our hospital, and four had unclear diagnoses at other hospitals and were transferred. Among these four patients, two had failed surgeries. After a definitive diagnosis was made during hospitalization, patients with a thin high-position transverse vaginal septum underwent successful resection. In contrast, two patients with a thicker high-position transverse septum experienced severe infection, fibrosis, and adhesion after septum resection, resulting in heavy bleeding at the surgical site. One patient was discharged after a 5-day hospital stay, while the other recovered and was discharged after 20 days.

**Figure 2:**
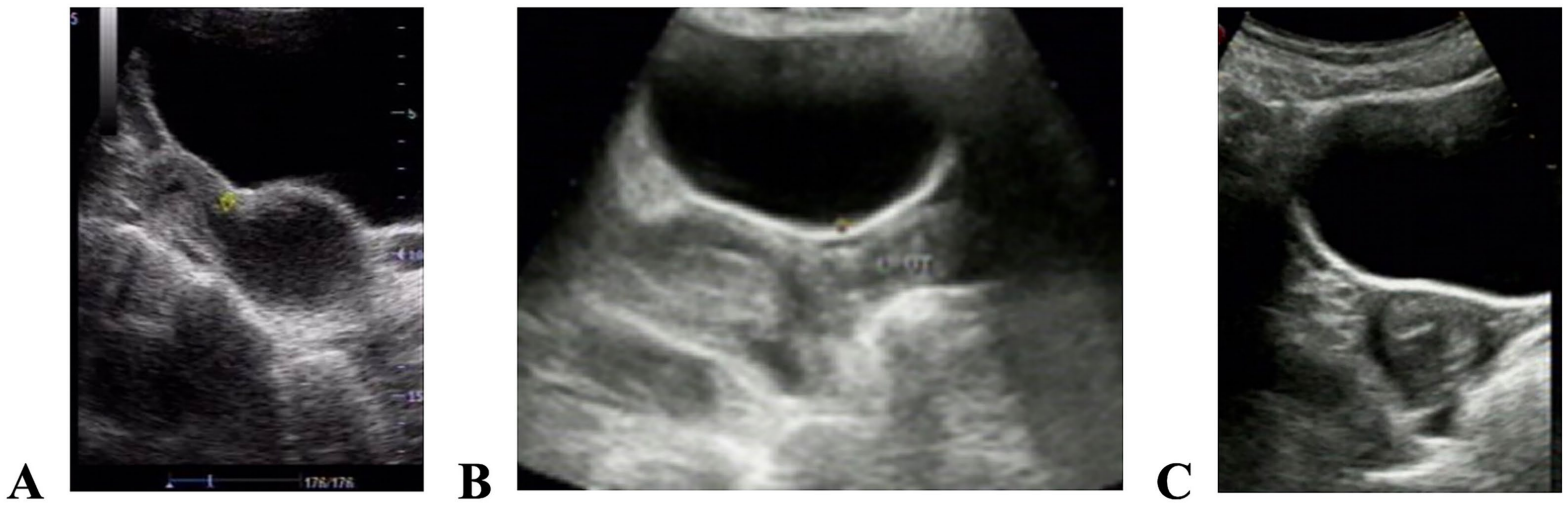
Pelvic ultrasound images before and after surgical correction of oblique vaginal septum syndrome, complicated by a rotated uterus. **(A)** Preoperatively, the right uterine myometrium showed a homogeneous echotexture, and separation of the uterine cavity was approximately 1.8 cm; an anechoic area measuring ~7.5 cm × 5.0 cm was detected inferior to the uterus, containing punctate weak echoes and communicating with the uterine cavity; **(B)** Postoperatively, the right uterine body measured ~4.5 cm × 2.2 cm and the left uterus measured ~3.9 cm × 2.0 cm; continuity between the endometrial line and the cervical canal was good bilaterally; on downward scanning along both uterine bodies, enlargement was observed at the confluence; **(C)** On transverse ultrasound scanning from the uterine body to the cervix and vagina, the two cervices showed a fused configuration inferiorly; the right cervix gradually shifted to the right anterior position, and the left cervix was located in the left posterior position.

A total of 13 cases with oblique vaginal septum syndrome (10) underwent vaginal septum resection and exposure of the affected side of the cervix, all of which were accompanied by ipsilateral renal agenesis. Among these 13 cases, one patient had a perforated vaginal septum with suspected pelvic lesions and therefore underwent laparoscopy, which revealed a tubo-ovarian abscess on the affected side. The abscess was resected, and pelvic drainage was placed, with complete resolution. Another case involved a right-sided imperforate vaginal septum, in which the cervical positions of the two uteri were misaligned (rotated uterus (11)) (Figure 2). The septum was removed, and the affected side of the cervix was exposed.

Uterus didelphys with unilateral type I cervical atresia, also known as oblique vaginal septum syndrome type IV, was identified in two cases: The patients had normal vaginal depth and width, both developed symptoms during adolescence, and had a history of surgery.

Case 1: A 22-year-old patient had first presented at the age of 14 with lower abdominal pain and underwent exploratory laparotomy in a local hospital, during which a “mass” was removed. After marriage, she experienced progressively worsening dysmenorrhea and pelvic masses. The local hospital diagnosed her with a pelvic abscess and attempted vaginal drainage, which was ineffective. In July 2007, she was transferred to our hospital. On gynecological examination, the cervix was shown to be located on the right side of the vaginal apex, which was underdeveloped, with a closed piercing scar on the left vaginal vault and a cystic, tender mass near the lower left side of the uterus. The uterus was normal in size but tilted to the right. Ultrasound examination showed no abnormalities in the kidneys. During surgery, an approximately 8 cm × 7 cm × 6 cm cystic, thick-walled mass was found on the left side of the uterus, fused with the right cervix, and could not be separated. Above the cystic mass, black thread-like structures were observed, suggesting a connection to the normal left adnexa. After drainage of the accumulated purulent blood from the closed end of the uterine isthmus through the vaginal incision, a left cervical plasty was performed by anastomosing the uterine isthmus to the vaginal apex. The patient was free of dysmenorrhea postoperatively.

Case 2: This patient had uterus didelphys with right lateral type I cervical atresia and right renal agenesis. She experienced abdominal pain during adolescence and underwent corrective surgery. Menstruation started thereafter; however, 3 months after her marriage, she presented with continuous large amounts of purulent vaginal discharge and was hospitalized. Surgical management involved expansion of the vaginal apex and the right-sided cervical blind end, and the two were anastomosed. A drainage tube was placed in the uterine isthmus postoperatively, and the patient was treated with anti-inflammatory therapy, with complete resolution (12). During surgery in both cases, tissue samples from the uterine isthmus cyst wall were sent for pathological examination, confirming the presence of uterine isthmus tissue.

**Figure 3:**
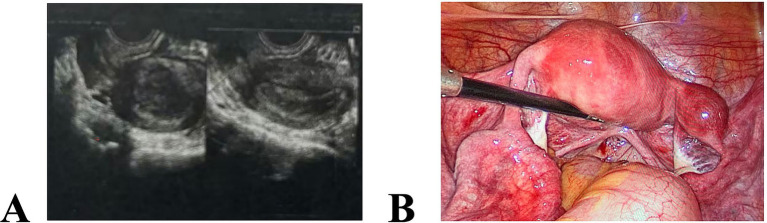
Preoperative pelvic ultrasound images and intraoperative findings of rudimentary horn pregnancy. **(A)** Preoperatively, the uterus was of normal size, and the endometrium was ~1.5 cm thick; a strip-like anechoic area approximately 0.2 cm in width was observed at the lower end of the uterine cavity. In the right adnexal region, medial to the right ovary, a predominantly solid mixed mass measuring ~2.3 × 2.0 cm was noted, with a small amount of cystic echoes; **(B)** Intraoperatively, a uterine body located slightly to the left in the pelvis was of normal size but abnormal in shape; on the right, a muscular mass approximately 3.5 cm in diameter was observed, with abundant surface blood supply and a violaceous-red color, and the two were connected by a muscular cord-like tissue.

There was one case of type II rudimentary horn uterus: The patient was 26 years old and had one prior normal vaginal delivery. She was admitted emergently for amenorrhea and lower abdominal pain, and ultrasonography indicated an ectopic pregnancy. Emergency laparoscopy revealed a uterine body located slightly to the left in the pelvis, of normal size but abnormal in shape; the left adnexa appeared normal. On the right, a muscular mass approximately 3.5 cm in diameter was noted, with abundant surface blood supply and a violaceous-red color; the two were connected by a muscular cord-like tissue. The right adnexa and the right round ligament were attached to the muscular mass, and the right adnexa was otherwise unremarkable. A right rudimentary horn pregnancy was considered, and right rudimentary horn resection and right salpingectomy were performed. During resection of the muscular cord-like tissue between the mass and the left uterine body, no obvious luminal structure communicating with the left uterine body was observed on the cut surface, consistent with type II rudimentary horn uterus (Figure 3). Postoperative pathology confirmed right rudimentary horn pregnancy.

## 3 Results

All 72 cases were accurately diagnosed, and after appropriate treatment, the outcomes were favorable. In 41 cases of 46,XX androgen excess type disorders of sex development, postoperatively, the external genitalia appeared nearly normal. During postoperative follow-up, 20 married patients reported improved sexual intercourse without notable difficulty. In 24 cases of incomplete vaginal obstruction, obstructive symptoms of 23 patients resolved postoperatively. One patient with a thicker, high-position vaginal septum developed high fever, abdominal pain, prolonged menstruation, and purulent vaginal discharge during menstruation 6 months postoperatively. Upon examination, the cervical opening at the vaginal apex was difficult to expose, suggesting inflammatory edema and hyperplasia at the vaginal apex surgical site, resulting in cervical canal narrowing, obstruction, and pelvic inflammation. Symptoms were alleviated after anti-inflammatory treatment. In two cases of uterus didelphys with unilateral cervical atresia, the patients underwent perineal-vaginal and cervical anastomoplasty, and menstruation became normal without recurrence of obstructive symptoms. In Case 1, the normal-sided uterus successfully supported two pregnancies.

## 4 Discussion

### 4.1 Incomplete obstructive diseases of the female genital tract and their classification

To increase the awareness of dysmenorrhea in adolescence and provide clinical practitioners with a more intuitive approach to diagnosis and treatment, we reviewed 72 cases accumulated at our hospital and relevant literature. Based on the location of the obstruction and clinical presentation, we classified female genital tract developmental abnormalities with abnormal menstrual blood discharge into four types: incomplete vaginal orifice obstruction, incomplete vaginal obstruction, incomplete cervical obstruction, and incomplete uterine cavity obstruction, which resulted in 24 types of diseases (Table 2).

**Table 2:** Classification of incomplete obstructive diseases of the female genital tract.

Classification	Site	Name of disease
Incomplete vaginal orifice obstruction	Vulva	Incomplete hymenal atresia (bridge-shaped, cribriform)
		46,XX androgen excess type disorders of sex development (Prader III, IV, V)
Incomplete vaginal obstruction	Vagina	Incomplete transverse vaginal septum (high-position, middle-position, low-position)
		Oblique vaginal septum syndrome (type I, type II, type III) (10) Communicating uterus (IIa, IIb, Va, Vb) (15)
Incomplete cervical obstruction	Cervix	Uterus didelphys with unilateral cervical atresia (type I, type IV) (Oblique vaginal septum syndrome type IV) Longitudinal cervical septum (2) Incomplete transverse cervical septum (16)
Incomplete uterine cavity obstruction	Uterine cavity	Oblique uterine septum (Robert uterus, incomplete oblique uterine septum) (17) Incomplete transverse uterine septum (18) Rudimentary horn of the uterus: Type I (Communicating uterus type X), type II (10)

Incomplete obstructive diseases of the female genital tract have important clinical significance in the differential diagnosis of unexplained dysmenorrhea in adolescents, helping to reduce delayed treatment or inappropriate management caused by misdiagnosis as “primary dysmenorrhea” or “pelvic inflammatory disease.” This classification is not intended to replace mainstream systems such as European Society of Human Reproduction and Embryology / European Society for Gynaecological Endoscopy (ESHRE/ESGE); rather, it further refines and supplements the “obstructive” subtypes within existing frameworks and is valuable for better addressing the clinical problem of “menstruation with obstructive symptoms.”

### 4.2 Clinical characteristics of incomplete obstructive diseases of the female genital tract

**Figure 4:**
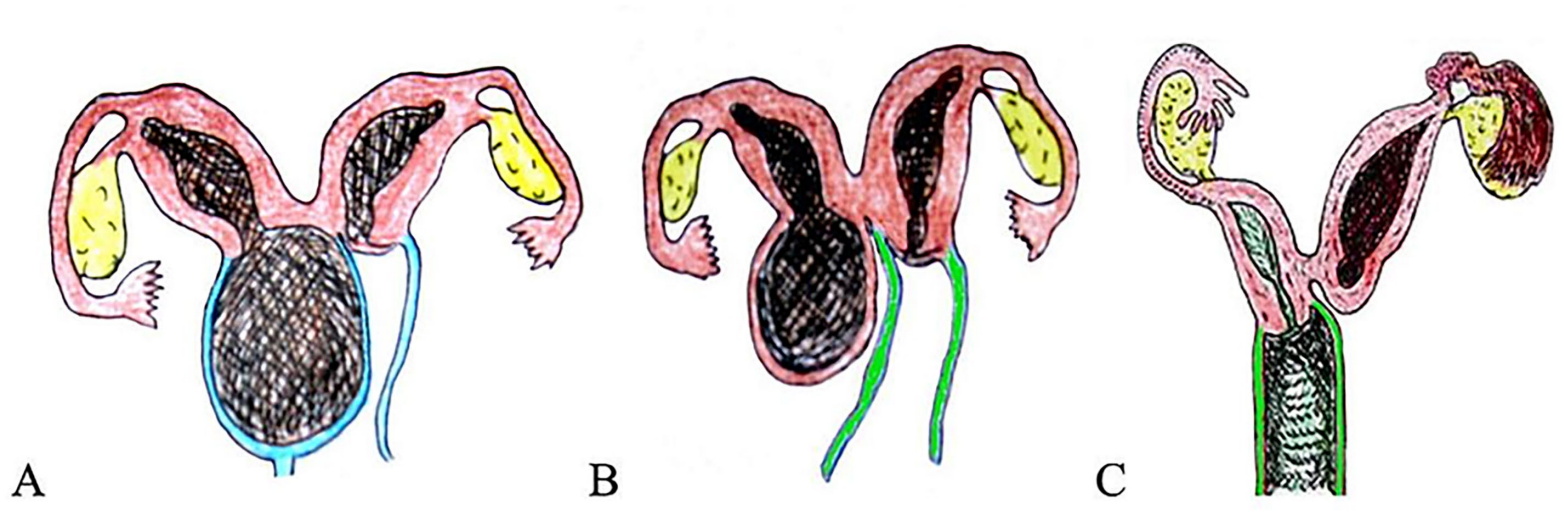
Schematic diagram of the differential diagnosis of oblique vaginal septum syndrome and uterus didelphys with unilateral cervical atresia. **(A)** The oblique vaginal septum has a posterior septal cavity, and the top of the posterior septal cavity has a basically normal developing cervix; **(B)** For uterus didelphys with unilateral cervical atresia (type I), it is necessary to open the top vaginal wall of the atresia side and the blind end of the cervical atresia during the correction surgery, but the two are anastomosed; **(C)** Uterus didelphys with unilateral cervical atresia (type IV), during surgical correction, the affected-side uterus may be resected, or the vaginal apex and the blind end of the cervix can be opened and then anastomosed, with short-term catheter placement in the uterine isthmus postoperatively.

All patients with female genital tract incomplete obstruction have normal menarche. Depending on the anatomical location and extent of the obstruction, clinical manifestations may include ① Obstruction: Obstructive symptoms such as prolonged menstrual period, dysmenorrhea, abnormal vaginal secretions, pelvic masses, abnormal development of the external genitalia, difficulty with sexual intercourse, challenges in inducing abortion, obstruction during childbirth, and abnormal development of the vulva; ② Complications: Infection symptoms such as abnormal vaginal discharge, abdominal pain, and fever, together with signs of pelvic endometriosis; and ③ Gynecological examination and imaging tests: Abnormal development of the external genitalia or genital tract. Among the 24 diseases identified so far, incomplete hymenal atresia, female pseudohermaphroditism, rudimentary horn of the uterus, and high- and mid-position incomplete transverse vaginal septum are relatively straightforward to diagnose, and the treatment methods are relatively simple. In contrast, incomplete transverse cervical septum, longitudinal cervical septum, and incomplete transverse uterine septum are rare. Diagnosis and corresponding treatment should be guided by clinical manifestations, imaging examinations, and intraoperative findings. Congenital oblique vaginal septum, owing to increased discussion in the literature, has led to greater professional understanding of this condition. Once a clear diagnosis is made, oblique vaginal septum resection can be performed to fully expose the posterior septal cavity and the cervix. For the following relatively rare cases, it is necessary to have a thorough understanding to avoid misdiagnosis and mistreatment, which could lead to more serious consequences for patients:

1. Oblique uterine septum is a rare, specialized septum within the uterine cavity, located on one side of the cavity. This septum is approximately 1.5 cm thick, generally originating from the upper right portion of the uterine cornua at the fundus of the uterus and extending diagonally downward, slightly above the internal os of the cervix on the same side, dividing the corpus uterine cavity into the upper-right and lower-left parts. The majority of oblique uterine septum cases are not associated with urinary system malformations and are divided into two types: ① Complete oblique uterine septum (also known as Robert uterus): In this type, the septum seals one side of the uterine cavity, and patients typically present with primary dysmenorrhea. Ultrasound examination of symptomatic patients may reveal the uterine septum along with blood accumulation on one side of the uterine cavity. Intraoperatively, the uterus appears enlarged with an asymmetrical saddle shape in some areas, which differs from the unicornuate cavity typically visualized on hysterosalpingography; and ② Incomplete oblique uterine septum: In this type, the septum is slanted downward, dividing the uterine cavity into two asymmetrical parts. The affected uterine cavity is smaller and situated superiorly, appearing as a small triangular cavity with an apex directed downward. There may be a minute foramen located in the inferior aspect of the septum. Since the menstrual blood is discharged without obstruction, the patient may have no symptoms. Complications such as dystocia or failed induced abortion may arise if pregnancy occurs in the affected cavity; in such cases, abnormal uterine development should be considered, and appropriate examinations should be conducted to confirm the diagnosis. An oblique uterine septum can be removed by hysteroscopic surgery under ultrasound guidance and laparoscopic monitoring.
2. Uterus didelphys with unilateral cervical atresia (type I and type IV) (oblique vaginal septum syndrome type IV): Cervical atresia is diagnosed using the classification proposed by Xie Zhihong et al. (13): type I is the incomplete cervical atresia, type II is the uterine isthmus atresia, type III is the complete cervical atresia, and type IV is the absence of the uterine isthmus. Patients with uterus didelphys with unilateral cervical atresia (type I or type IV) have one side of the uterus developing normally while the cervix on the other side has atresia. The depth and width of the vagina are normal, but the top of the vagina on the atresia side also has atresia. Patients have normal menstruation but, because menstrual blood from the affected side cannot be discharged, they may experience severe dysmenorrhea and other symptoms, which are easily misdiagnosed as type I–III oblique vaginal septum syndrome. The surgical treatment methods for the two are substantially different (Figure 4).
  For patients with a congenital oblique vaginal septum, there is a posterior vaginal septal cavity, and at the top of the posterior septal cavity, there is a basically normally developed cervix. Removing the septum to expose the cervix can solve the problem. For patients with uterus didelphys with unilateral type I cervical atresia, a stoma can be made at the top of the perineum vagina and the blind end of the cervix and then the two can be anastomosed to preserve the uterus. For uterus didelphys with unilateral type IV cervical atresia, the affected uterus can be removed, or the vaginal apex and the blind end of the cervix can be opened and then the two can be anastomosed to preserve the uterus. After the operation, a catheter is placed in the cervical canal for a short period until the formed cervical wall is fully epithelialized. It is not advisable to get pregnant in the future because the absence of the isthmus of the uterus may lead to miscarriage (12).
  Notably, some patients with uterus didelphys may experience secondary dysmenorrhea due to underdevelopment of the endometrium on the affected side, which can occur a significant period after menarche.
3. High-position incomplete transverse vaginal septum: In cases of congenital high-position incomplete transverse vaginal septum, menstruation occurs on time during puberty. However, if the septum opening is small, particularly after marriage, menstrual blood may not flow smoothly because of vaginal inflammation and edema, resulting in symptoms such as irregular menstruation and other signs of infection. Over time, this can result in the formation of inflammatory granulation tissue and fibrosis on the septum, worsening menstrual retention symptoms and, in some cases, progressing to complete obstruction. Pelvic inflammatory symptoms may also develop.

For patients with a generally normal vaginal depth but in whom the cervix cannot be visualized, this condition should be considered, and imaging studies should be performed to confirm the diagnosis. For those with a “thicker” incomplete vaginal septum, during surgery, the vaginal septum and the inflammatory proliferative connective tissue between the septum and cervix should be removed. Postoperatively, iodoform gauze should be used to apply pressure to the vaginal surgical site to control bleeding, and effective antibiotics should be administered until the inflammation subsides and the wound is fully epithelialized before discharge. This approach helps prevent postoperative inflammatory hyperplasia of the vaginal apex tissue, which could lead to cervical stenosis and potential re-obstruction (14).

Treatment-related complications and long-term reproductive outcomes: ① Risk of restenosis: Restenosis may occur after various reconstructive procedures (vaginoplasty and cervicoplasty), requiring long-term monitoring; ② Risk of infection: After the relief of obstruction, particularly in patients with a history of hematometra/pyometra, the risk of postoperative pelvic infection has increased; therefore, perioperative prophylactic antibiotics should be emphasized; and ③ Reproductive risk: In women with malformations, such as unicornuate uterus and Robert uterus, even after relief of obstruction, conception rates, miscarriage rates, and preterm birth rates may remain higher than in women without malformations. In patients who undergo reconstructive surgery for unilateral cervical atresia, attention should be paid to the risk of cervical insufficiency during pregnancy. This information is important for long-term management and fertility counseling.

### 4.3 Occurrence of incomplete obstruction of the female genital tract

The development and differentiation of the female genital tract is a complex and delicate process. During development, abnormalities in the Müllerian ducts or paramesonephric ducts can lead to various genital tract malformations, with diverse clinical presentations. Clinically, some patients experience menarche but may have abnormal menstrual discharge pathways, along with symptoms such as scanty or irregular menstrual flow and more severe dysmenorrhea due to obstruction.

Based on the clinical data of female genital tract developmental abnormalities with abnormal menstrual blood discharge, obstructive malformations can be further subdivided into complete and incomplete obstructions. The occurrence of incomplete obstruction can be due to the following four conditions:

1. Developmental incomplete obstruction of the genital tract.
2. Abnormal fusion and resorption of bilateral paramesonephric ducts: One side of the genital tract remains patent while the other side becomes obstructed.
3. Abnormal development of the urogenital sinus: This may result in masculinization of the external genitalia or, combined with narrowing of the lower vaginal segment, may result in abnormal menstrual blood discharge pathways and sexual intercourse difficulties.
4. Iatrogenic causes: Incomplete obstruction can result from improper treatment of congenital complete genital tract obstructions. For example, the two cases of high-position incomplete transverse vaginal septum in this study were caused by this factor.

In Case 1, the patient had no menarche and suffered from cyclic lower abdominal pain for 3 months before being surgically treated at a local hospital. Menstruation started after surgery, but it was later diagnosed that the patient actually had a congenital high-position complete transverse vaginal septum, and the surgery only created a small opening, converting the complete septum to an incomplete one.

In Case 2, after 3 years of periodic abdominal pain, the patient was treated at a local hospital, and menarche began. However, the diagnosis of congenital high-position complete transverse vaginal septum was unclear due to the absence of menarche and a pelvic mass (blood accumulation in the vaginal septum cavity pushing the uterus upward). After exploratory laparotomy, menstrual blood from the obstructed vaginal cavity was evacuated through the raised vaginal blind end, which inadvertently resulted in a high-position incomplete transverse vaginal septum.

Similarly, in cases of uterus didelphys with unilateral cervical atresia, if the diagnosis and treatment are inappropriate, it can lead to incomplete patency or re-obstruction of the cervical canal on the affected side. The two cases of uterus didelphys with unilateral type I cervical atresia reported in this study experienced dysmenorrhea and pelvic masses during adolescence and underwent surgery at local hospitals. In Case 1, the excised “mass” was the uterine corpus, which relieved the symptoms. However, after marriage, sexual stimulation caused the residual endometrial function in the excised uterus to restore, resulting in the recurrence of menstrual retention symptoms. In Case 2, after the cervical blind end was opened during adolescence, chronic retained menstrual blood was temporarily expelled, but due to incomplete cervical patency, ascending infection occurred.

### 4.4 Limitations of the study

This study has several limitations. First, this was a single-center retrospective study with a relatively limited sample size (*n* = 72), and all cases were from the same hospital, introducing a risk of selection bias. A comprehensive classification may therefore not be achievable. Accordingly, we referenced previous literature when classifying diseases in order to pursue a classification that is as comprehensive as possible, but categories not included in the classification may still exist. Second, retrospective data collection may result in incomplete or inconsistent clinical information, e.g., detailed physical examination records and standardized symptom scores. Third, for subjective outcomes such as pain, sexual function, and quality of life, we were unable to apply validated standardized scales within a retrospective framework, which may have affected the objectivity of the results. Future studies should adopt prospective designs and multicenter collaboration and incorporate validated patient-reported outcome measures to provide higher-level evidence.

In summary, the concept of “incomplete obstructive diseases of the female genital tract” proposed in this study along with the four-type classification based on the site of obstruction (incomplete obstruction at the vaginal orifice, the vagina, the cervix, and the uterine cavity) provide a systematic synthesis of the pathological spectrum underlying the distinctive clinical presentation of menarche accompanied by obstructive symptoms. This classification framework is intended to assist clinicians, particularly those evaluating refractory dysmenorrhea in adolescents, by offering clearer diagnostic guidance. For patients who have experienced menarche but present with dysmenorrhea or other clinical manifestations of genital tract obstruction, incomplete obstructive diseases of the female genital tract should be considered, and appropriate examinations should be performed, thereby avoiding delays in diagnosis and treatment and improving long-term prognosis.

## Data Availability

To protect patient confidentiality, raw data cannot be shared on public platforms in compliance with privacy and ethical restrictions set by the hospital’s ethics committee. However, de-identified data may be provided upon reasonable request, contingent upon appropriate ethical approval. Interested parties may contact the corresponding author via email for further inquiries.
